# Maclise’s Surgical Anatomy

**Published:** 1850-11

**Authors:** 


### ARTICLE I.

#### MACLISE'S SURGICAL ANATOMY.

The third part of this magnificent work is devoted to the anatomy and surgery of Inguinal and Femoral Hernia. One more number completes the work, which, when correspondingly bound, will be the most splendid volume issuing from the American medical press for the current year. The price of the whole will be eight dollars. A moderate price, all things considered. We hope the eminent and enterprising publishers may meet with a sale sufficient to re-imburse them for their trouble and expenditure. M.

